# Surgical treatment of benign prostatic hyperplasia: Thulium enucleation versus single‐port transvesical robotic simple prostatectomy

**DOI:** 10.1002/bco2.261

**Published:** 2023-06-22

**Authors:** Susan Talamini, Andrew Lai, Cassandra Palmer, Gabriel van de Walle, Marcin Zuberek, Simone Crivellaro

**Affiliations:** ^1^ Washington University School of Medicine St. Louis Missouri USA; ^2^ College of Medicine University of Illinois at Chicago Chicago Illinois USA

**Keywords:** BPH, single‐port robotic prostatectomy, thulium laser enucleation

## Abstract

**Objective:**

The objective of this work is to compare our outcomes using thulium laser enucleation of prostate (ThuLEP) to the single‐port robot‐assisted simple prostatectomy (SP RASP) in the surgical management of benign prostatic hyperplasia (BPH).

**Methods:**

A retrospective cohort study was conducted from January 2017 through December 2021 of men who underwent SP RASP and ThuLEP performed by a single surgeon with an enucleation experience of >300 cases and extensive robotic experience. The primary outcome was changed in International Prostate Symptom Score (IPSS) postoperatively. Secondary outcomes were operative time, length of stay (LOS), change in post‐void residuals (PVR), de novo stress‐ or urge‐urinary incontinence (SUI, UUI), and rate of complications.

**Results:**

One hundred two patients underwent surgery during the study period: 33 RASP and 69 ThuLEP. There was no difference in preoperative characteristics, including age and body mass index, between both groups. Changes in IPSS scores postoperatively were not significant between SP RASP versus ThuLEP (−17 vs. −14, *p* = 0.2956). SP RASP had a longer operative time (180 vs. 90 min, *p* < 0.0001). There was no difference in LOS (0 vs. 0 days, *p* = 0.2904). There was no difference in change in PVR (−96 vs. −91 mL, *p* = 0.8504). SP RASP patients had significantly less postoperative SUI than ThuLEP (0 vs. 13 patients, *p* = 0.0083), while there was no difference in UUI between both groups (4 vs. 2 patients, *p* = 0.0843). There was no difference in 30‐day complication rate (21.2% vs. 21.7%, *p* = 0.9517), although there were three ThuLEP patients with Clavien–Dindo Class III or higher complication.

**Conclusions:**

There was no difference in change in IPSS scores between the two groups. ThuLEP is associated with shorter postoperative catheter days and decreased operative times. Hospital LOS was equivalent. SP RASP demonstrates significantly improved continence rates. Though SP RASP is within the initial learning curve at our institution, early results demonstrate the role for this modality alongside ThuLEP in the treatment of large gland BPH.

## 1 INTRODUCTION

Benign prostatic hyperplasia (BPH) is a common affliction in men, and prevalence increases with age with some 70% of men 61–70 years old experiencing pathological BPH.^1^, ^2^ The implications of clinical BPH can be significant, leading to decreased quality of life and well‐being secondary to bothersome lower urinary tract symptoms (LUTS).^3^, ^4^, ^5^ Ultimately, severe bladder outlet obstruction (BOO) due to BPH may lead to recurrent urinary tract infections (UTI), acute and chronic urinary retention, development of bladder stones, and upper tract deterioration, and surgical intervention may be required.^2^ In the surgical management of patients with large (80–150 g) to very large (>150 g) glands, the American Urological Association (AUA) recommends consideration of simple prostatectomy (open, laparoscopic, or robotic), and laser enucleation may be considered as a prostate size‐independent option as well.^6^

Transurethral laser enucleation of the prostate for BPH has been performed using thulium laser enucleation of prostate (ThuLEP) and holmium laser enucleation of prostate (HoLEP) energies and has been demonstrated to be safe, cost effective, and provide durable long term functional outcomes for all size glands.^7^, ^8^, ^9^, ^10^, ^11^, ^12^ Comparison studies between the two modalities appear to demonstrate similar outcomes, and therefore choice of laser energy should be per surgeon preference.^13^, ^14^, ^15^ Our institution prefers the use of the thulium laser for prostate enucleation, and this study will focus primarily on this energy modality.

The da Vinci single‐port (SP) robotic platform (Intuitive Surgical, Sunnyvale, CA) received FDA approval for urologic use in 2018, and since that time, multiple studies have been published evaluating its utility and outcomes in major urologic procedures, including prostatectomy, ureteral reimplantation, donor nephrectomy, and transvesical operations.^16^, ^17^, ^18^, ^19^, ^20^, ^21^ Our institution has largely abandoned the use of a multiport (MP) approach to the robotic simple prostatectomy in favour of the SP system owing to its narrow profile, articulating camera, and facility in operating in a confined space. Additionally, our adoption of a novel docking system has made cystotomy needed for intravesical access less than 2 cm in size, further reducing the invasiveness of the procedure.

This study aims to compare our experience and patient functional outcomes using ThuLEP to the SP robotic‐assisted simple prostatectomy (SP RASP).

## 2 METHODS

### 2.1 Study design and patient population

A retrospective cohort study was conducted between January 2017 through December 2021. All surgeries were performed by a single surgeon with an enucleation experience of over 300 cases along with extensive robotic experience. All patients who underwent either ThuLEP or SP RASP for benign prostatic enlargement or urinary retention due to prostatic obstruction with a minimum of 1 month follow‐up were eligible for inclusion in the study. Patients were excluded if they were lost to follow‐up after surgery. This study was performed in alignment with the guidelines on Strengthening the Reporting of Observational Studies in Epidemiology (STROBE).^22^

The ThuLEP and SP RASP surgeries were respectively performed in two different hospitals with similar catchment populations. This was due to the resource capabilities at each hospital. The operative teams were well trained in their respective hospitals. The surgeon operated at both hospitals with full privileges. Patients at both institutions with indications for surgery were offered choices of surgical approach and decided based on shared decision making. This study received approval from the institutional review board (IRB) from both institutions.

### 2.2 Evaluation of outcome measures

The primary outcome was change in International Prostate Symptom Score (IPSS) postoperatively. Secondary outcomes were operative time, length of stay (LOS), change in post‐void residuals (PVR), de novo stress‐ or urge‐urinary incontinence (SUI, UUI), and 30‐day complications by Clavien–Dindo classification.^23^ Descriptive data were also collected. All data were collected through review of the electronic medical records. Operative time was discerned from anaesthesia and nursing records. Indications for surgery included bothersome LUTS, BPH, catheter‐dependent urinary retention secondary to bladder outlet obstruction. Preoperative measurements of prostatic enlargement were not routinely performed prior to ThuLEP, given the prostate size‐independent indication for use of this treatment modality.^6^ SP RASP was offered to patients with large and very large prostates, as evaluated on cystoscopy, MR, abdominal ultrasound, and CT scan as many of these imaging modalities had been performed as a component of the workup. Follow‐up for these patients were variable based on patient's postoperative symptoms and outcomes, as there was no protocol in place at the time of the study.

### 2.3 Statistical analysis

Using SAS® Studio 2022 (SAS Institute Inc., Cary, NC), Wilcoxon‐Mann–Whitney test for hypothesis tests on continuous variables was performed. Chi‐square or Fisher exact tests were used for categorical variables. A statistical significance level (*α*) of 0.05 was used.

## 3 TECHNIQUE

### 3.1 SP RASP

The patient is placed supine on the operating table on a no‐slip Pink Pad (Xodus Medical, New Kensington, PA), and the arms are tucked. An 18Fr Foley is placed on the field and the bladder emptied. A flexible cystoscope is introduced per the urethra with CO_2_ gas insufflation. A vertical 3‐cm incision is made 2–3 fingerbreadths above the pubic bone as previously described.^24^ The dissection is carried down to the fascia, which is then opened vertically and the rectus muscle is split bluntly, opening the retropubic space. The fat is then cleared from the bladder. The light from the cystoscope aids in the dissection. The bladder detrusor is then opened to create a mucosal diverticulum and four 0‐vicryl stay sutures are placed and an approximately 2‐cm cystotomy is made. The 18 Fr Foley catheter is replaced.

A wound protector component of the SP access port is placed into the bladder and a 5‐mm assistant port is placed to the right side of the bladder under palpation for utilization of rigid suction. The SP access port is attached to the wound protector component, and then the robot is docked using the floating technique with a 5‐mm AirSeal inserted through the side port for insufflation. The robot is then docked.

Ureteral orifices are visualized. The dissection line is marked circumferentially. We then begin to enucleate the prostate by developing a plane with electrocautery along the anterior bladder neck at the level of the surgical plane between peripheral part and adenoma. Once this is well established, we proceed to develop the lateral aspect of the plane bilaterally, with care taken to avoid the ureteral orifices (UOs). We then focus on the posterior plane. The surgical plane is developed from the bladder neck to the apex. The enucleation plane is further developed circumferentially until the prostate apex is reached. We then transect the urethral mucosa cold, releasing the prostate adenoma. Pressure is then dropped to 8 mmHg, and haemostasis is obtained.

A posterior reconstruction is performed using a 3‐0 V‐Loc suture, incorporating the urethra, posterior peripheral zone, and the bladder neck. The bladder neck reconstruction is performed using a 3‐0 double armed Stratafix suture, with the goal of collapsing the prostatic fossa created from the enucleation. A 20 Fr 3‐way haematuria catheter is placed, and the balloon is inflated with 20‐cc sterile water.

The cystotomy is closed in two layers with running 2‐0 vicryl for the mucosa and the 0 vicryl stay sutures for the second layer. Patients are discharged the same day, and the Foley catheter is routinely removed on postoperative day (POD) 5.

### 3.2 Enucleation

The patient is placed in the high dorsal lithotomy position and a 24Fr sheath resectoscope with a visual obturator is used initially. The obturator is removed and a 30° lens is inserted. Bilateral ureteral orifices are identified.

A 1000‐μm thulium fibre (Quanta Systems, Samarate VA, Italy) is used, with coagulation and vaporization settings adjusted for the prostate at 30 W. Settings can also be adjusted to a pulse or continuous mode, with the pulse setting providing shallower tissue penetration. Our institution prefers an en‐bloc enucleation technique. Care is taken while initially developing the enucleation plane to perform an early release of the sphincter complex to prevent traction injury. This is performed by defining the enucleation plane circumferentially proximal to the sphincter by marking and releasing the mucosa first. The verumontanum is used as the distal marker for resection and a deeper U‐shaped marking is made just proximal to this. The plane is started at the six o'clock position proximal to the verumontanum, and then carried out laterally at the three and nine o'clock position, and anteriorly at the 12 o'clock in a retrograde fashion. This circumferential dissection releases the sphincter complex and prevents prolonged traction on the sphincter during the blunt dissection required during the enucleation. Following this, the lateral planes are developed partially. The anterior plane is developed until the bladder is entered at the anterior bladder neck. This plane is then carried down and connected with the lateral planes until the entire adenoma is released and then pushed into the bladder.

A Piranha morcellator (Richard Wolf Medical Instruments Corp, Vernon Hills, IL) is used to remove the adenoma. It is crucial to maintain constant bladder pressure and volume during morcellation to aid in visibility and to prevent bladder injuries. Our institution uses the Thermedx FluidSmart Fluid management system (Stryker Corp., Kalamazoo, MI). Pressures of 60 mmHg are maintained during the enucleation component, and 130 mmHg during the morcellation. Upon completion of morcellation, a 22Fr 3‐way haematuria catheter is inserted, and balloon filled with 80‐cc saline, and placed on light traction which is released after approximately 1 h. Patients typically are discharged the same day with the catheter and return to the clinic for catheter removal in 3–4 days.

## 4 RESULTS

A total of 103 patients underwent surgery during the study period: 34 SP RASP and 69 ThuLEP. One patient was lost to follow‐up from the SP RASP group. The patient demographics and preoperative characteristics are summarized in Table 1. There were no statistical differences between the two groups. The median follow‐up time for the RASP and ThuLEP cohort was 4.5 and 5.0 months, respectively (*p* = 0.3929).

**TABLE 1:** Patient demographic and preoperative clinical characteristics (*N* = 102).

	RASP (*N* = 33) median (IQR) or *N* (%)	ThuLEP (*N* = 69) median (IQR) or *N* (%)	*p* value*
Age in years	68 (64–72)	68 (63–74)	0.9401
Body mass index in kg/m^2^	28.2 (24.4–31.2)	28.1 (25.9–31.3)	0.7579
ASA score			0.7885
I	0	1 (1.9%)	
II	14 (42.4%)	19 (36.5%)	
III	19 (57.6%)	32 (61.5%)	
Preoperative medications			0.1955
α‐antagonist	13 (39.3%)	37 (54.5%)	
α‐antagonist + 5ARI	19 (57.6%)	26 (38.2%)	
None	1 (3.0%)	5 (7.4%)	
Catheter dependent	14 (42.4%)	32 (46.4%)	0.7074
PVR in mL	120 (24–300)	180 (48–500)	0.1713
IPSS	26.5 (20–29)	22 (14–31)	0.4074

Abbreviations: 5ARI, 5‐α‐reductase inhibitor; ASA, American Society of Anesthesiology; IPSS, International Prostate Symptom Score; IQR, interquartile range; PVR, post‐void residual; RASP, single‐port robot‐assisted simple prostatectomy; ThuLEP, thulium laser enucleation of prostate.

* Mann–Whitney *U* test, chi‐squared test, or Fisher's exact test; *α* = 0.05.

Table 2 summarizes the postoperative clinical outcomes. There was no difference in change in IPSS score between the SP RASP and ThuLEP cohorts (−17 vs. −14, *p* = 0.2956). There were no conversions to open surgery. There was no difference in LOS (*p* = 0.2904). The final prostate specimen weight was larger in the SP RASP cohort (74 vs. 42 g, *p* = 0.0106). There were no SP RASP patients postoperatively who developed SUI (0 vs. 13, *p* = 0.0083), while there was no difference in number of patients who developed postoperative UUI (4 vs. 2, *p* = 0.0843). There was no difference in the proportion of patients who had any 30‐day complications (21.2% vs. 21.7%, *p* = 0.9517). Operative times were significantly longer for SP RASP versus ThuLEP (180 vs. 90 min, *p* < 0.0001). SP RASP patients had significantly longer catheter time than ThuLEP (6 vs. 3 days, *p* < 0.0001).

**TABLE 2:** Summary of clinical outcomes (*N* = 102).

	RASP (*N* = 33) median (IQR) or *N* (%)	ThuLEP (*N* = 69) median (IQR) or *N* (%)	*p* value*
Follow‐up time in months	4.5 (2.0–9.0)	5.0 (1.0–21.0)	0.3929
Prostate specimen weight in grams	74 (46–87) [maximum 213]	42 (24–78) [maximum 157]	0.0106
Change in IPSS	−17 (−23 to −8)	−14 (−20 to −11)	0.2956
Length of stay in days	0 (0‐1)	0 (0‐0)	0.2904
Postoperative SUI	0 (0%)	13 (18.8%)	0.0083
Postoperative UUI	4 (12.1%)	2 (2.9%)	0.0843
Pathology			
BPH	28	55	
Prostate cancer	5	14	
GG1	2	11	
GG2	2	1	
GG3	1	0	
GG4	0	1	
GG5	0	1	
Operative time in minutes	180 (164–270)	90 (70–300)	*p* < 0.0001
Catheter time in days	6 (5–7)	3 (3–6)	*p* < 0.0001

Abbreviations: GG, Gleason grade group; IPSS, International Prostate Symptom Score; IQR, interquartile range; RASP, single‐port robot‐assisted simple prostatectomy; SUI, stress urinary incontinence defined as ≥1 pads per day; ThuLEP, thulium laser enucleation of prostate; UUI, urge urinary incontinence.

* Mann–Whitney *U* test, chi‐squared test, or Fisher's exact test; *α* = 0.05.

There were no significant difference in the proportion of any 30‐day complication between the SP RASP and ThuLEP (21.2% vs. 21.7%, *p* = 0.9517) (Table 3). There were three major complications in the ThuLEP group. One patient experienced clot retention requiring takeback to the operating room. One patient required extended continuous bladder irrigation for over 24 h and sustained acute renal failure. The third patient experienced sepsis secondary to urinary tract infection leading to acute renal failure, which required dialysis. The SP RASP group did not experience any major complications. The difference in the category of classification was not significant (*p* = 0.1016).

**TABLE 3:** Summary of complications (*N* = 102).

	RASP (*N* = 33) *N* (%)	ThuLEP (*N* = 69) *N* (%)	*p* value*
Any 30‐Day Complications	7 (21.2%)	15 (21.7%)	0.9517
Clavien Dindo I	4 (12.1%)	8 (11.6%)	
Clavien Dindo II	3 (9.1%)	4 (5.8%)	
Clavien Dindo IIIb	0	1 (1.4%)	
Clavien Dindo IVa	0	2 (2.9%)	0.1016

Abbreviations: RASP, single‐port robot‐assisted simple prostatectomy; ThuLEP, thulium laser enucleation of prostate.

* Chi‐squared test, or Fisher's exact test; *α* = 0.05.

## 5 DISCUSSION

The transurethral resection of prostatic (TURP) adenoma has long been considered the gold standard in the treatment of BPH. However, performing bipolar or monopolar TURP when approaching large or very large glands may be challenging due to longer operative times, bleeding risk, and likelihood of incomplete resection of tissue. Advances in technology have allowed us to revisit old techniques, that is, open simple prostatectomy, but offer less morbid, minimally invasive, and durable responses for all size prostates. To our knowledge, this is the first study comparing outcomes between ThuLEP and the SP RASP.

While simple prostatectomy has been utilized for many years, it has evolved from an open approach to the preferred robotic approach. This minimally invasive approach decreased morbidity by reducing blood loss, hospital stay, and pain.^25^ The SP robot further reduces the morbidity of the procedure.^16^

Previous studies have demonstrated that both laser enucleation of the prostate and SP RASP provided durable and comparable improvement in symptoms of BPH. A recent systematic review suggested the enucleation be the preferred approach due to decreased EBL, catheter days and length of hospital stay.^26^, ^27^ However, a multicentric study evaluating outcomes of SP RASP demonstrated that mean EBL was 100 cc, catheter days were approximately 5 days, though this was variable between institutions performing this procedure. Additionally, this study noted the practice of same day discharge after SP RASP.^28^ Our institution routinely performs same day surgery for these patients, and patients are discharged with minimal to no narcotics. Further analysis of these practices may be considered to address patient satisfaction and associated costs.

The utilization of the purpose‐built SP access port system allows for improved use of the “floating dock technique.” This provides increased working surgical space, in particular in small surgical fields with a close surgical target.^28^, ^29^ Furthermore, the access port allows for a smaller cystotomy as the size of the cystotomy need only accommodate the Alexis retractor component of the access port, and the combined width of the instruments, which enter the bladder with a combined diameter of less than 2.5 cm. Additionally, the port allows for rapid instrument exchanges, suture exchange, and specimen retrieval. We recognize that the docking of the robot and closing of the incision leads to longer operative times. The longer catheter times with SP RASP is attributable to surgeon's preference to allow for the cystotomy to heal.

While ThuLEP may be considered less invasive as compared to SP RASP, as no incision is required, the avoidance of urethral instrumentation during SP RASP should be considered as well. The vigorous movements during enucleation risks formation of urethral and meatal strictures. Incidence of urethral and bladder neck strictures after ThuLEP or HoLEP range from 1 to 5%, and while this risk was significantly greater for smaller glands in these studies, this risk can be reduced with a transvesical approach due to almost complete avoidance of urethral instrumentation.^30^, ^31^

Many laser enucleation scopes commonly used during enucleation are 26 or 28 Fr in size, though our institution utilizes the 24Fr Wolf scope. The 24 Fr scope ideally reduces the urethral trauma secondary to instrumentation as compared with larger scopes. An ongoing clinical trial aims to further evaluate the impact of this reduced diameter scope on vision, flow rates, and bladder injuries.^32^

The complication rates between two groups have not been statistically different; however, there were more Clavien III and higher complications in the THULEP group. These were related to postoperative bleeding associated with specimen morcellation and clot retention, which required intraoperative clot evacuation and bleeding control. Both stem from the imprecise nature of laser enucleation and morcellation of the specimen inside of the bladder. Although the number of patients in the SP RASP group is smaller, in our experience, the patients are not brought back to the operating room for clot evacuation and bleeding control as the instruments used for SP RASP allow for a more precise enucleation plane and better directed haemostasis. The authors acknowledge that more long‐term follow‐up is necessary capture any delayed complications.

Our study identified a significant difference in rates of SUI, with SP RASP showing decreased rates of incontinence. We attribute this to the ability to perform a bladder neck reconstruction, moving the urethra back in the pelvic space and better control over the apical dissection. The collapse of the fossa obtained with this reconstruction also aids in haemostasis, obviating the need for continuous bladder irrigation and therefore hospital admissions.

Our study is not without limitations. Both surgeries were performed by a single surgeon at a high‐volume academic centre. We recognize that our experience may not be generalizable to the community setting. Additionally, our data do not directly compare our outcomes to those from open simple prostatectomies. The retrospective component of our study presents difficulty with variable follow‐up. We did not have a standardized follow‐up protocol or pathway for either surgical cohort. Additions of flow parameters preoperatively and postoperatively would be another objective measurement to assess functional outcomes.

## 6 CONCLUSION

There was no difference in change in IPSS score between SP RASP and ThuLEP. ThuLEP is associated with shorter postoperative catheter days and decreased operative times. Hospital LOS was equivalent. SP RASP demonstrates significantly improved continence rates, likely a consequence of the bladder neck reconstruction and possibly reduced traction injury to the sphincter complex. While previous studies have recommended use of laser enucleation ahead of RASP in the treatment of large glands due to shorter LOS and reduced blood loss, this study adds to the evidence suggesting there is equivalent LOS between the modalities, due to the practice of same day discharge with the SP platform. The use of SP RASP is relatively new at our institution, though early results demonstrate the role for this modality alongside ThuLEP in the treatment of large gland BPH.

## AUTHOR CONTRIBUTIONS

All contributions have been made with authorship in the manuscript.

## CONFLICT OF INTEREST STATEMENT

Dr. Simone Crivellaro is a consultant for Intuitive Surgical, Inc. All other authors have nothing to disclose.
